# Imagery adds stimulus-specific sensory evidence to perceptual detection

**DOI:** 10.1167/jov.22.2.11

**Published:** 2022-02-17

**Authors:** Nadine Dijkstra, Peter Kok, Stephen M. Fleming

**Affiliations:** 1 Wellcome Centre for Human Neuroimaging, Queen Square Institute of Neurology, University College London, London, UK; 2 Wellcome Centre for Human Neuroimaging, Queen Square Institute of Neurology, University College London, London, UK; 3 Wellcome Centre for Human Neuroimaging, Queen Square Institute of Neurology, University College London, London, UK; 4 Max Planck UCL Centre for Computational Psychiatry and Aging Research, University College London, London, UK; 5 Department of Experimental Psychology, University College London, London, UK

**Keywords:** mental imagery, reality monitoring, perception

## Abstract

Internally generated imagery and externally triggered perception rely on overlapping sensory processes. This overlap poses a challenge for perceptual reality monitoring: determining whether sensory signals reflect reality or imagination. In this study, we used psychophysics to investigate how imagery and perception interact to determine visual experience. Participants were instructed to detect oriented gratings that gradually appeared in noise while simultaneously either imagining the same grating, a grating perpendicular to the to-be-detected grating, or nothing. We found that, compared to both incongruent imagery and no imagery, congruent imagery caused a leftward shift of the psychometric function relating stimulus contrast to perceptual threshold. We discuss how this effect can best be explained by a model in which imagery adds sensory signal to the perceptual input, thereby increasing the visibility of perceived stimuli. These results suggest that, in contrast to changes in sensory signals caused by self-generated movement, the brain does not discount the influence of self-generated sensory signals on perception.

## Introduction

Visual signals can be triggered externally, by the appearance of a stimulus in the outside world (i.e., perception), or internally, via top-down processes such as mental imagery. Neuroimaging evidence has shown that neural representations of internally and externally generated signals are highly similar (Dijkstra, Bosch, & van Gerven, 2019; Horikawa, Tamaki, Miyawaki, & Kamitani, 2013; Pearson, 2019). This overlap might complicate perceptual reality monitoring: determining whether a given sensory signal reflects external reality or internal imagination (Dijkstra, Kok, et al., 2022). In order to understand how the brain is able to keep imagined and perceived signals separate, it is necessary to gain insight into how internally and externally generated sensory signals interact to determine visual experience.

One line of research has shown that mental imagery can bias perception of subsequent ambiguous stimuli toward the imagined stimulus (Pearson, 2014; Pearson, Clifford, & Tong, 2008) and that the strength of this effect is proportional to the vividness of the imagery (Bergmann et al., 2016; Keogh et al., 2020; Keogh & Pearson, 2014). However, results obtained during simultaneous imagery and perception, a situation in which perceptual reality monitoring is particularly challenging, have been more mixed. Early studies found that simultaneous imagery *decreased* the likelihood that participants detected external stimuli, which was interpreted as showing that the external input was erroneously being attributed to imagery (Perky, 1910; Segal & Fusella, 1969; Segal & Glicksman, 1967; Segal & Nathan, 1964). This line of evidence suggests that during imagery, the brain explains away or suppresses incoming sensory signals. However, an imagery-induced increase in misses could also be due to distraction or decreases in (sensory) processing capacity (Craver-Lemley & Reeves, 1992; Reeves et al., 2020; Segal & Fusella, 1970). In contrast, later experiments reported that when the same stimulus was simultaneously imagined and perceived, participants were *more likely* to report perceptual presence (Dijkstra & Fleming, 2021; Dijkstra, Mazor, et al., 2021; Farah, 1985, 1989; Moseley et al., 2016; Saad & Silvanto, 2013). These results have been interpreted as showing that imagined signals are incorporated into perception (Dijkstra, Mazor, et al., 2021). These latter observations are inconsistent with the brain explaining away or suppressing sensory signals when engaging in imagery and instead suggest that imagined signals are combined with incoming sensory signals, increasing the probability of reporting external stimulus presence.

**Figure 1.**
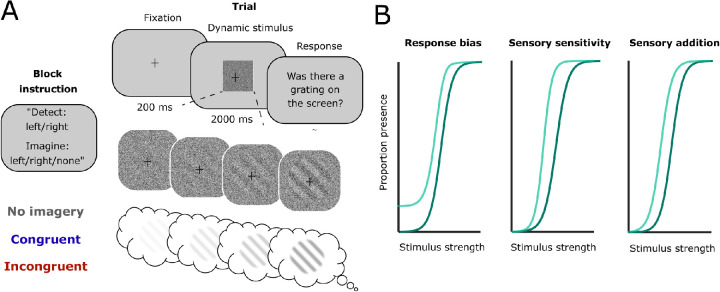
Experimental design and hypotheses. (A) Experimental paradigm. At the start of each block, participants were instructed whether they would be detecting left- or right-tilted gratings and what they had to imagine this block: nothing, a left-tilted grating, or a right-tilted grating. Trials consisted of 200-ms fixation followed by 2 s of dynamically fluctuating noise in which a stimulus gradually ramped up until a certain visibility level. The task was to indicate whether a grating was present or not. After each block, participants were asked what they had imagined during this block to check whether they accurately followed instructions. (B) Hypotheses. Imagery might increase or decrease perceptual presence response via different mechanisms. Left: a response bias would be reflected in a change in guess rate and mean. Middle: a change in sensory sensitivity would be reflected in a change in slope. Right: a subtraction or addition to the sensory signal would be reflected in only a change in mean.

Increases or decreases in reports of perceptual presence—a change in detection criterion—can be caused by several different underlying mechanisms, not all of them reflective of changes in sensory processing (Gold & Ding, 2013; Witt et al., 2015). Within a signal detection theoretic framework, a criterion shift can be due to a change in response bias, a shift in the underlying sensory signals, or both (Wyart, Nobre & Summerfield, 2012). To shed light on this question, here we quantified the effects of imagery on the psychometric function for detection to dissociate three hypothesized mechanisms for how simultaneous imagery and perception interact. Participants detected tilted gratings that gradually appeared in noise and simultaneously imagined the same grating, a grating perpendicular to the to-be-detected grating, or nothing (Fig. 1A). Importantly, stimulus contrast was varied parametrically, allowing us to estimate the full psychometric function relating stimulus strength to the probability of detecting an external grating in each imagery condition (Wichmann & Hill, 2001a, 2001b).

We hypothesized that imagery could lead to an increase or decrease in perceptual presence responses via different mechanisms (Fig. 1B). First, simultaneous imagery might cause a response bias in favor or against the imagined stimulus, without changing sensory processing. This would be reflected by a vertical shift of the psychometric curve for the congruent condition, quantified by a change in both the mean and the guess rate—as such a response bias should cause an increase in presence responses independently of stimulus contrast. Furthermore, such a stimulus-independent response bias should be observed most prominently at low contrast values (equivalent to classical false alarms) and then be washed out by an increasing influence of the stimulus as contrast increases (Fig. 1B, left). Second, top-down imagery might change the sensitivity to external sensory signals by amplifying signals proportionally to their strength. This would be reflected by a change in both the slope (variance) and the mean of the psychometric curve (Reynolds & Heeger, 2009; Fig. 1B, middle). Finally, imagined signals might simply be added or subtracted from the perceived signal, reflected by a horizontal shift in the psychometric curve, associated most strongly with a change in the mean and potentially with a small change in guess rate if the curve does not converge to zero presence responses at zero contrast (Fig. 1B, right).

## Methods

### Participants

Based on power calculations of a previous experiment with the same paradigm (Dijkstra, Mazor, et al., 2021), we required 34 participants to capture differences in detection criterion and accuracy between conditions. Given that we are interested in slightly more subtle effects in the current study, we decided to recruit 50 participants. Participants were recruited online via Prolific (www.prolific.co), and data were collected on an institutional server managed by the JATOS tool (Lange et al., 2015). All participants gave written informed consent to participate in the study, and all procedures were approved by the University College London ethics committee. The experiment took approximately 40 min to complete, and participants were paid £5 for their participation, equivalent to an hourly rate of £7.50. Data from two participants were not obtained due to technical issues. Furthermore, we excluded five participants because they failed the imagery check (see below) and an additional two because of poor psychometric curve fits. The final sample consisted of 41 participants (mean age = 29.14 years, *SD* = 11.5).

### Experimental procedure

The experimental paradigm is depicted in Figure 1A. The experiment was programmed in JavaScript using jsPsych (de Leeuw, 2015). Participants were instructed to detect gratings that gradually appeared in dynamic noise while simultaneously imagining the same grating (congruent condition), a grating perpendicular to the presented grating (incongruent), or nothing (no imagery). Prior to the main experiment, participants filled out the VVIQ2 (Marks, 1995), which we used to instruct participants what we meant by the concept of mental imagery. After this, participants first practiced detecting gratings with a high contrast until they responded correctly on at least 75% of the trials, making sure they understood the task. Then, participants practiced imagining the gratings while looking at the dynamic noise for 20 trials in a row, 10 per orientation. They were instructed to imagine the grating “as if it was actually presented.” After each trial, participants were asked to indicate the vividness of their imagery using a scale from 1 (*not vivid at all*) to 5 (*perfectly clear and as vivid as real seeing*), similar to the scale used in the VVIQ2.

For the main task, participants were instructed that a grating of one orientation would be presented on some of the trials and that in some cases, the grating would be quite hard to see. In order to avoid visual priming, no trial-wise cues were delivered, and instead the different conditions were implemented in a block-wise fashion such that during the entire block, participants detected one specific orientation and imagined one specific orientation. At the onset of each experimental block, the participant was instructed which grating orientation would be shown and what they had to imagine during the block (Fig. 1A) as follows: “During this block you will see right/left tilted gratings. Please do not/also imagine left/right-tilted gratings as vividly as possible.” To start with a block, participants had to press the space bar. There were 12 blocks, two per condition (orientation × imagery), each consisting of 42 trials. The order of the blocks was randomized within each participant. Each trial started with a 200-ms fixation cross followed by 2 s of either pure dynamic white noise or dynamic white noise within which a gradually appearing stimulus was embedded. The task of the participants was to indicate whether or not a grating was present on each trial. After each block, participants were asked which, if any, stimulus they had imagined during this block, to ensure that they had correctly followed the instructions. Blocks were removed prior to analyses if the answer to this imagery check was incorrect.

At the end of the experiment, after filling out their age, we also asked participants whether they thought imagining the gratings had altered their responses on the detection task. The replies varied, with comments such as “I think imagining either left or right tilted gratings helped me see the ones that were actually there” and “I made some mistakes in the answers because I got confused between the pattern that I imagined and the one that was displayed.”

### Stimuli

The stimuli were generated in MATLAB (version R2018b; The MathWorks, Natick, MA, USA) and consisted of a sinusoidal grating at an orientation of 45^o^ or 135^o^ masked with an annulus and embedded in white noise (Fig. 1A). The visibility of the stimulus indicated the probability that a given pixel contained the grating stimulus rather than noise (Mazor et al., 2020), that is, the percentage signal relative to noise. Seven visibility levels were used: 0%, 3.7%, 4.8%, 5.3%, 6.1%, 7.3%, and 14% (Fig. 1B). The exact values of the visibility levels were predetermined via piloting to allow for accurate estimation of the psychometric curves.

For each orientation separately, 50 stimulus images were generated and distributed equally in log space across the seven visibility levels (from 0% to 14%). For the pure noise trials, an additional 20 images of pure white noise were generated. For the trials with a visibility level above zero, 20 stimulus images ranging from zero visibility to that specific visibility level were presented over the course of 2 s, giving the impression that the stimulus was gradually appearing in the noise. This ramping up was done to mimic the gradual nature of mental image generation (Perky, 1910). During zero visibility trials, 20 noise images were presented in a random order.

### Data analysis

Per imagery condition, we fit the detection responses for the separate visibility levels using the following cumulative gaussian function:

$$P_{present}=g+\left(1-g\right)\frac{1}{2}\left[1+\mathrm{erf}\left(\frac{x-\mu }{\sigma \sqrt{2}}\right)\right]$$

where *P_present_* is the probability of reporting stimulus presence;  μ is the mean of the normal distribution, reflecting the horizontal offset of the psychometric curve/how much signal is needed to achieve 50% presence responses; σ is the standard deviation, reflecting the slope or sensitivity of presence responses to increases in signal; and *g* is the guess rate, reflecting the vertical offset at the zero point, or how likely presence responses are in the complete absence of signal. We did not include a lapse rate (Wichmann & Hill, 2001a) because we did not have prior hypotheses about this parameter, and therefore including it would unnecessarily increase the complexity of the model. Fitting was performed using maximum likelihood via the binomial link function. The curve parameters μ and σ were initialized at 0.05 (5%), and *g* was bounded to be between 0 and 1 by transforming it to another parameter, θ, such that $g=\;\frac{1}{\left(1+e^{-\theta }\right)}$, where θ was initialized at −8, corresponding to a guess rate close to zero. Statistical inference was performed by comparing the three curve parameters between conditions using analyses of variance.

## Results

**Figure 2.**
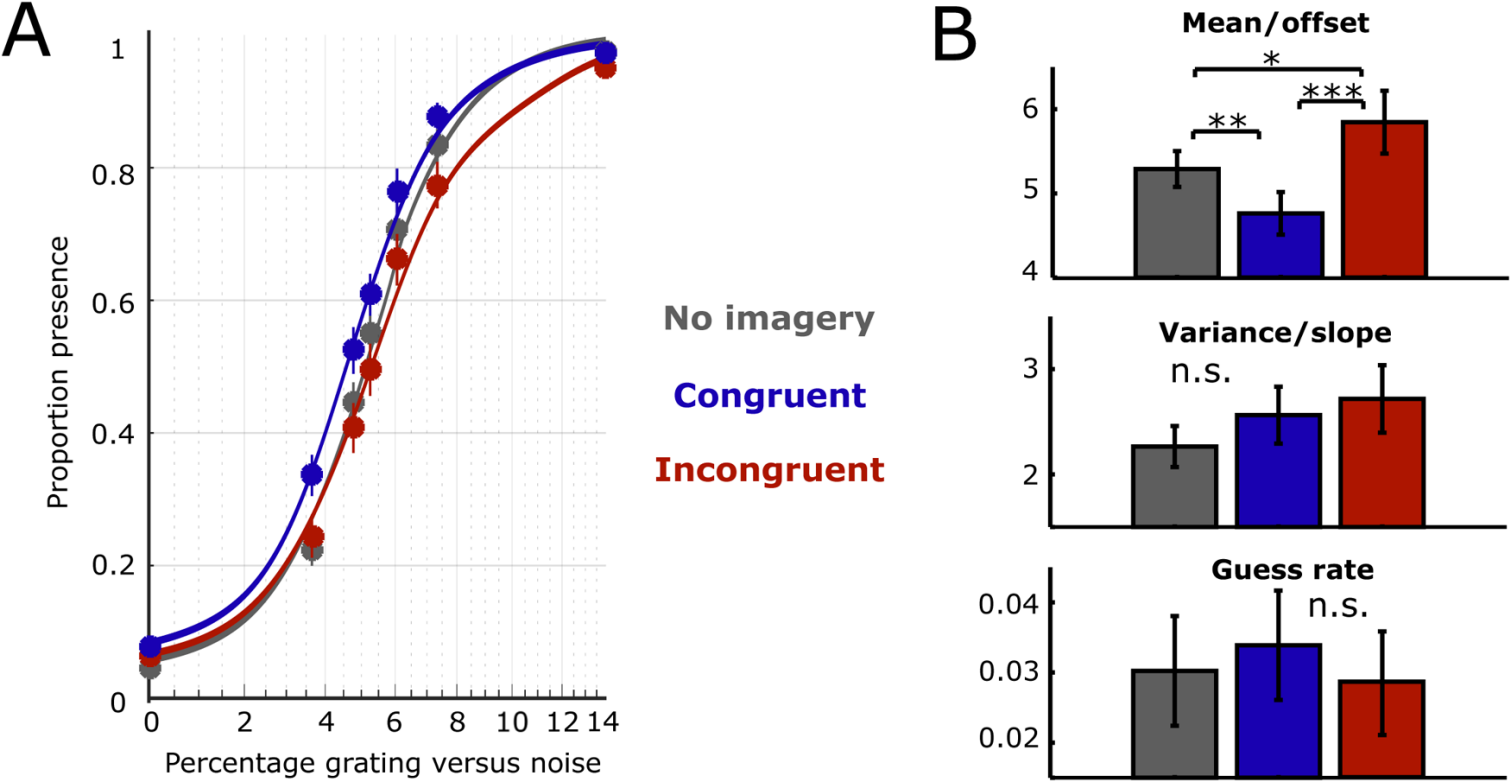
Results. (A) Psychometric function per condition. Solid points represent proportion presence trials at the different visibility levels for each condition averaged over participants. Vertical lines reflect *SEM* for these points. (B) Curve parameter estimates. Top: mean, the signal value that is associated with a 0.5 probability of reporting stimulus presence. Middle: variance/slope, the sensitivity of presence responses to changes in signal. Bottom: guess rate, the proportion of presence responses for zero signal. *p < 0.05; **p < 0.01; ***p < 0.001.

The curve fits and proportion of presence responses per visibility level for the three conditions are shown in Figure 2. Mental imagery caused a horizontal shift of the psychometric curve, reflected in a significant effect of condition on the mean (Fig. 1C; *F*(39, 2) = 7.34, *p* = 0.002, η_p_^2^ = 0.273). Post hoc comparisons revealed that the amount of signal needed to achieve 50% presence responses was significantly lower during congruent imagery (*M* = 4.76, *SD* = 1.61) than during no imagery (*M* = 5.29, *SD* = 1.36; *t*(40) = 2.97, *p* = 0.005) or during incongruent imagery (*M* = 5.85, *SD* = 2.39; *t*(40) = 3.63, *p* = 0.0007). Furthermore, the mean was also significantly higher during incongruent imagery compared to no imagery (*t*(40) = 2.12, *p* = 0.038). In contrast, there was no significant effect of imagery condition on either the slope (*F*(39, 2) = 1.325, *p* = 0.277) or the guess rate (*F*(39, 2) = 0.169, *p* = 0.845). We further quantified the evidence for the presence or absence of a condition difference using Bayesian statistics in JASP (JASP & JASP Team, 2019). In keeping with the interpretation of Bayes factors from Jeffreys (1961) and Lee and Wagenmakers (2014), there was extreme evidence for an effect on mean (*BF* = 109.85), moderate evidence for the absence of an effect on slope (*BF* = 0.29), and strong evidence for the absence of an effect on guess rate (*BF* = 0.08).

## Discussion

In this study, we set out to characterize how internally and externally generated visual signals interact when stimuli are simultaneously imagined and perceived. To this end, we estimated psychometric functions while participants were simultaneously detecting and imagining oriented gratings. We found that congruent imagery was associated with a leftward shift of the psychometric function compared to both no imagery and incongruent imagery. In contrast, imagery did not have an effect on either the slope or the guess rate of the psychometric function. These results suggest that imagery adds sensory evidence to perceptual signals, thereby increasing the visibility of perceived stimuli.

Imagery did not influence the guess rate of the psychometric function. This makes it unlikely that imagery led to a response bias toward reporting stimulus presence, for instance, due to demand characteristics. Such a bias in the context of our experiment might have caused participants to blindly respond “yes” more often during congruent imagery, even in the absence of any changes in sensory processing (Dijkstra, Mazor, et al., 2021). However, if this were the case, it would have led to an increase in presence responses across the full range of stimulus strengths and therefore also a shift in the guess rate parameter. In contrast, we found that the influence of imagery on perceptual detection was best captured by a change in the horizontal offset without a change in guess rate, indicating imagery interacts with sensory input such that the effect of imagery was largest at intermediate levels of sensory evidence.

While the absence of an effect on guess rate rules out a simple response bias, a change in mean could be caused by effects at different levels of sensory processing. The effect could be due to either a change in low-level sensory representations and/or by a change in the decision variable in more high-level evidence accumulation circuits (Gold & Ding, 2013). Previous neuroimaging research has shown that imagery activates perception-like neural representations in low-level sensory areas (Albers, Kok, Toni, Dijkerman, & De Lange, 2013; Dijkstra, Bosch, & van Gerven, 2017; Lee, Kravitz, & Baker, 2012; Ragni, Tucciarelli, Andersson, & Lingnau, 2020), even in the presence of external stimuli (Rademaker et al., 2019). Furthermore, the imagery-induced horizontal shift in the psychometric function reported here is similar to previously observed effects of microstimulation of stimulus-preferred sensory neurons (Fetsch et al., 2014). Therefore, we believe that the most likely explanation for our findings is that imagery adds sensory signals to a perceptual input stage, creating an intermixed sensory representation, which is strongest when the imagined and perceived content match.

The precise implementation of how exactly imagery adds to the sensory signal remains unclear. One intriguing possibility is that imagery, like expectation, not only enhances activity of neural populations congruent with the imagined stimulus (Wyart et al., 2012) but also *suppresses* activity of incongruent populations—thereby “sharpening” the neural representation in line with imagination (Kok et al., 2012). This idea could neatly explain the pattern of results observed here: an increase in perceptual presence responses for congruent imagery would be due to enhancement of stimulus-specific activity, whereas incongruent imagery would instead suppress stimulus-specific activity (and enhance incongruent activity), thereby decreasing the probability of detecting the stimulus. This would suggest that expectation and imagery rely on similar neural mechanisms, in line with previous proposals (Feuerriegel et al., 2021; Pearson, 2019). Future research should further investigate the neural implementation of imagery's influence on perception and to what extent it is similar to effects of expectation.

The consistent increase in perceptual presence responses for congruent imagery reported here and elsewhere (Dijkstra, Mazor, et al., 2021; Farah, 1989; Moseley et al., 2016) suggests that the brain does not account for self-generated sensory signals during imagery. This observation seems to be in contrast to analogous mechanisms hypothesized to discount self-generated changes in sensory input caused by action. During action, the nervous system is thought to send a copy of the motor signal to sensory areas, leading to cancellation of the expected sensory change (Crapse & Sommer, 2008). The better the change in sensory input can be predicted by the movement, the stronger the agent has the feeling that it is self-generated rather than being caused by a change in the outside world (Haggard, 2017). However, recent behavioral and neuroimaging studies have found evidence for enhancement rather than suppression of self-generated, expected sensory consequences of actions (Dogge et al., 2018; Yon et al., 2018, 2020), raising doubts about whether sensory attenuation is necessary to distinguish self-generated from externally generated sensory signals during action. One important open question is how these effects of enhancement and suppression are related to source attribution. It is possible that these two effects (sensory attenuation and self-attribution) are dissociable—for instance, whether or not sensory changes are inferred to be self-generated may still be determined by the predictability of sensorimotor consequences, even if attribution to self is associated with an enhancement rather than a suppression of the sensory signals. This would mean that sensory signals predicted from self-generated actions would be more likely to be perceived than unexpected ones, in line with recent findings (Yon et al., 2020) and seemingly in line with the current results.

However, there is an important difference between reality monitoring of sensory signals during action and reality monitoring during imagery. During action, the sensory signals are always assumed to reflect real changes in external input—either caused by our own movement or by changes in the outside world. In other words, reality monitoring during action is not about whether there is a change in the external visual input of our hand but about whether that change is caused by us moving our hand or by some outside source. In contrast, reality monitoring during imagery involves a lower-level decision about whether there was *any* change in external visual input or whether such signals were generated by imagination—that is, in the current experiment, answering the question, “Was there a grating presented on the screen?” Answering yes to this question already implies that the sensory signal has an external source and was not imagined. In other words, attributing sensory changes during action to self can be associated with an increase in perceptual presence responses, whereas attributing sensory changes during imagery to self should be associated with a decrease in perceptual presence responses. Such decreases have been observed in the past (Perky, 1910), but recent evidence suggests that these effects might have been specific to historical context (Dijkstra & Fleming, 2021). A failure to correct for self-generated visual signals during imagery might play a role in reality monitoring errors causing hallucinations (Dijkstra, Kok, et al., 2022). More research is necessary to further investigate the relationship between self-attribution and changes in perceptual detection during imagery versus action.

## Conclusions

Taken together, our results suggest that congruent imagery increases the probability of reporting perceptual presence by adding sensory evidence in favor of the imagined stimulus. These findings indicate that the brain does not discount the impact of self-generated sensory signals generated during imagery on perception, instead treating it as additional evidence for the presence of external input.
